# Lipocalin-2 levels in children with idiopathic short stature

**DOI:** 10.1007/s12020-025-04437-y

**Published:** 2025-09-27

**Authors:** Naama Fisch-Shvalb, Meytal Bar-Maisels, Michal Yackobovitch-Gavan, Moshe Phillip, Galia Gat-Yablonski

**Affiliations:** 1 Institute of Endocrinology and Diabetes, Schneider Children’s Medical Center, Petach Tikva, Israel; 2 The Gray Faculty of Medical and Health Sciences, Tel Aviv University, Tel Aviv, Israel; 3 Epidemiology and Preventive Medicine, School of Public Health, The Gray Faculty of Medical and Health Sciences, Tel Aviv University, Tel Aviv, Israel

**Keywords:** Lipocalin-2, Idiopathic short stature, Height SDS, Growth

## Abstract

**Purpose:**

Idiopathic short stature (ISS) is defined as height below − 2 standard deviation scores (SDS) without identifiable causes. Lipocalin-2 (LCN2), a protein involved in inflammation and growth plate regulation, was hypothesized to be elevated in ISS, potentially disrupting growth via inflammatory or non-inflammatory mechanisms at the growth plate. This study aimed to compare serum LCN2 levels in children with ISS versus healthy controls and assess LCN2 as a biomarker for growth failure.

**Methods:**

In a case-control study (July 2023–January 2025), serum LCN2 levels were measured using ELISA in 28 pre-pubertal children with ISS (height ≤ 3rd percentile, normal growth hormone response) and 30 healthy controls (height ≥ 25th percentile). Height SDS, body mass index (BMI) SDS, and insulin-like growth factor-I (IGF-I) SDS were assessed.

**Results:**

Serum LCN2 levels were significantly lower in the ISS group (80.48 ± 20.78 ng/mL) compared to controls (95.37 ± 22.70 ng/mL; *p* = 0.012). Height SDS positively correlated with LCN2 levels (*r* = 0.264, *p* = 0.045), but no correlations were found with age, BMI SDS, or IGF-I SDS. BMI SDS was lower in the ISS than in the control group (-0.32 ± 0.81 vs. 0.35 ± 1.21; *p* = 0.015).

**Conclusions:**

Serum LCN2 levels may be lower in children with ISS than in children with normal stature. Future studies should attempt to elucidate the possible role of LCN2 in regulation of linear growth.

## Introduction

Linear growth is a complex process influenced by genetics, general health, nutrition and hormones. Short stature in childhood, defined as height below − 2 standard deviations (SD) for age, sex, and population, is a common reason for referral to pediatric endocrinologists, and can impact quality of life. Idiopathic short stature (ISS) is a diagnosis of exclusion, when identifiable causes—such as systemic diseases, endocrine disorders, genetic abnormalities, or nutritional deficiencies—have been ruled out. Recent genetic studies indicate that 25–40% of children with ISS have variants in genes related to the growth hormone/insulin-like growth factor-I (GH/IGF-1) axis or growth plate (GP) maturation [1, 2]. However, as these variants account for only a minority of cases, the etiology of ISS remains largely unknown [3].

Lipocalin-2 (LCN2), also known as neutrophil gelatinase-associated lipocalin, is a protein secreted by adipocytes, neutrophils and osteoblasts, with roles in iron transport, inflammation modulation, and metabolic regulation [4]. In the skeleton, LCN2 is expressed in chondrocytes during endochondral ossification [5–7] and in the bone tissue [8].

In a study conducted on LCN2-transgenic mice, animals overexpressing LCN2 showed reduced skeletal size, body weight, and femur length compared to wild-type (Wt) mice, starting from 2 to 3 weeks of age [7]. Histological staining of neonatal and 2-week-old mice growth plate sections found that both the proliferating and the hypertrophic chondrocyte zones were less organized in transgenic (Tg) than in Wt mice. Immunohistochemical staining for type II collagen was more intense and for type X collagen less intense in the femurs of LCN2-Tg mice. These findings indicate impaired chondrocyte maturation and proliferation in LCN2-Tg mice, contributing to delayed endochondral ossification and reduced skeletal size. Similarly, in a different rat model, daily systemic administration of LCN2 (150 ng/g) for 20 or 40 days resulted in downregulation of *Collagen X* and *RUNX2* at the growth plate chondrocytes indicating inhibition of chondrocyte hypertrophy via systemic exposure to LCN2 [9].

LCN2 expression in growth plate chondrocytes is also increased after exposure to dexamethasone, and has been suggested to mediate glucocorticoid-induced growth plate dysfunction and growth retardation [6]. Its expression is also elevated after exposure to pro-inflammatory factors such as IL-1 and lipopolysaccharide [10]. Thus, at the growth plate, LCN2 may mediate the negative effects of both glucocorticoids and inflammation on linear growth.

A previous proteomic analysis of serum from children with ISS compared with controls found higher serum LCN2 levels compared with healthy controls, suggesting that serum LCN2 could serve as a biomarker to distinguish children with ISS from those with normal height [9]. Given its suggested roles in growth plate regulation, this study aimed to compare serum LCN2 levels in children with ISS versus healthy controls and to assess whether elevated serum LCN2 levels are associated with growth failure in ISS.

## Materials and methods

### Study design

This case-control study compared serum LCN2 levels in children with ISS and healthy controls with normal height. The study was conducted at Schneider Children’s Medical Center, Israel, between July 2023 and January 2025, and was approved by the Rabin Medical Center Institutional Review Board (study number RMC-0127-23). All parents provided written informed consent.

### Participants

#### Study group

Girls and boys aged 3–9 years with ISS were recruited from those referred for growth hormone (GH) stimulation testing due to short stature, as per consensus guidelines [11]. Inclusion criteria were: (1) pre-pubertal status (Tanner stage 1), (2) height ≤ 3rd percentile for age and sex per Centers for Disease Control and Prevention (CDC) norms, and (3) peak GH response >7.5 ng/mL on stimulation testing, confirming normal GH secretion. Exclusion criteria included: (1) small for gestational age at birth, (2) chronic diseases or morbidity, (3) GH deficiency, (4) treatment with recombinant human GH, (5) chronic medications affecting growth or appetite, and (6) acute illness within 1 week of recruitment. GH stimulation tests used glucagon, arginine, clonidine, or combined clonidine-arginine [12], performed after an overnight fast between 08:00 and 11:00 by trained staff.

## Control group

Healthy, pre-pubertal (Tanner stage 1) boys and girls aged 3–9 years with height ≥ 25th percentile for age and sex (CDC norms) were recruited as controls. Participants were either volunteers (e.g., children of healthcare workers) or patients undergoing minor elective surgical procedures (e.g., inguinal herniotomy, skin tag removal, ventilation tube insertion). Exclusion criteria mirrored those of the study group: (1) chronic diseases, (2) chronic medications affecting growth or appetite, and (3) acute illness within 1 week of recruitment. Blood samples were collected after anesthesia but before surgery, following an overnight fast, between 08:00 and 11:00.

### Assays

Serum LCN2 levels were measured using a commercial enzyme-linked immunosorbent assay (ELISA) kit (Human Lipocalin-2/NGAL Quantikine QuicKit, Cat. No. QK1757; R&D Systems, Minneapolis, MN, USA), following the manufacturer’s instructions. Serum GH concentrations were determined by chemiluminescent enzyme immunoassay on an Immulite Automated Analyzer (Diagnostic Products Corp), calibrated to the international standard 98/574 using GH Adjustors Calibra Liquid (Bio-Rad Laboratories). A peak GH response > 7.5 ng/mL on stimulation testing excluded GH deficiency (GHD). Serum IGF-1 levels were measured using an immunoassay kit on a Liaison Analyzer (DiaSorin), with IGF-1 standard deviation scores (SDS) calculated based on manufacturer reference intervals.

### Data collection

Anthropometric measurements (height, weight, body mass index [BMI]) were performed by a pediatric endocrinologist or trained nurse at enrollment. Pubertal stage assessment (Tanner method, based on genital status in boys and breast development in girls) was performed by a pediatric endocrinologist. BMI was calculated as weight (kg) divided by height (m²) and expressed as SDS per CDC recommendations [13]. Medical history, chronological age, parental height (study group only), and GH stimulation test type were extracted from medical records.

### Statistical analysis

Sample size was calculated assuming a clinically significant difference of 30 ng/mL in plasma LCN2 levels between groups [9] with a standard deviation of 27 ng/ml [14], requiring at least 14 participants per group for 80% power at a 5% significance level. Accordingly, 30 children with ISS and 30 controls were recruited. Data normality was assessed using histograms, Q-Q plots, and the Kolmogorov-Smirnov test. Group differences were evaluated using independent sample t-tests (normal distribution) or Mann-Whitney U-tests (skewed distribution). Categorical data were compared using chi-square or Fisher’s exact tests. Correlations between LCN2 levels and anthropometric/biochemical parameters (e.g., IGF-1 SDS) were analyzed using Pearson’s (normal distribution) or Spearman’s (skewed distribution) correlation coefficients. A linear regression model was used to examine the effect of the group (ISS vs. control) on mean serum LCN2 levels after adjustment for BMI SDS. Analyses were performed using SPSS version 29.

## Results

### Participant Characteristics

 Between July 2023 and January 2025, 64 children were enrolled. Six were excluded due to GHD: five with confirmed GHD and one where GHD could not be ruled out. The final cohort comprised 28 children with ISS and 30 controls (8 healthy volunteers and 22 before undergoing minor surgical procedures). Baseline characteristics are presented in Table 1. Median age was similar between groups (ISS: 6.83 years, interquartile range [IQR] 4.42–7.66; controls: 5.37 years, IQR 3.98–6.70; *p* = 0.077), as was male sex distribution (ISS: 46%; controls: 53%; *p* = 0.599). Median height SDS was significantly lower in the ISS group (-2.29; IQR − 2.66 to -2.03) than in controls (0.29; IQR − 0.18 to 0.90; *p* < 0.001). Mean BMI SDS was lower in the ISS group (-0.32 ± 0.81) compared to controls (0.35 ± 1.21; *p* = 0.015). Based on CDC BMI percentiles, 27/28 ISS children had normal weight (BMI < 85th percentile), and 1 was overweight (BMI 85th–95th percentile). In controls, 18 had normal weight, 6 were overweight, and 6 were obese (BMI ≥ 95th percentile), with a higher prevalence of overweight/obesity in controls (*p* = 0.003). For the ISS group, mean mid-parental height SDS was − 1.11 ± 0.63, mean stimulated GH peak was 12.00 ± 3.69 ng/mL, and mean IGF-1 SDS was 0.11 ± 0.63.

**Table 1:** Baseline characteristics and serum LCN2 levels in children with idiopathic short stature (ISS) and controls

Variable	ISS (*n* = 28)	Control (*n* = 30)	*P*-value
Age, years, median (IQR)	6.83 (4.42, 7.66)	5.37 (3.98, 6.70)	0.077
Male sex, n (%)	13 (46)	16 (53)	0.599
Height SDS, median (IQR)	-2.29 (-2.66, -2.03)	0.29 (-0.18, 0.90)	**< 0.001**
BMI SDS, mean ± SD	-0.32 ± 0.81	0.35 ± 1.21	**0.015**
LCN2 levels, ng/ml, mean ± SD	80.48 ± 20.78	95.37 ± 22.70	**0.012**
MPH SDS, mean ± SD	-1.11 ± 0.63	NA	
Stimulated GH peak, ng/ml, mean ± SD	12.00 ± 3.69	NA	
IGF-1 SDS, mean ± SD	0.11 ± 0.63	NA	

BMI body mass index, GH growth hormone, IGF-1 insulin-like growth factor-1, IQR interquartile range, LCN2 Lipocalin-2, MPH mid-parental height, SDS standard deviation score, NA not applicable

### Serum LCN2 levels

Mean LCN2 levels were significantly lower in the ISS group (80.48 ± 20.78 ng/ml) compared to the control group (95.37 ± 22.70 ng/ml), *P* = 0.012. In a linear regression model, the difference in mean LCN2 levels between the ISS and the control group persisted after adjustment for BMI SDS (B = 15.6, SE = 6.08, *P* = 0.013).

### Correlations

**Fig. 1:**
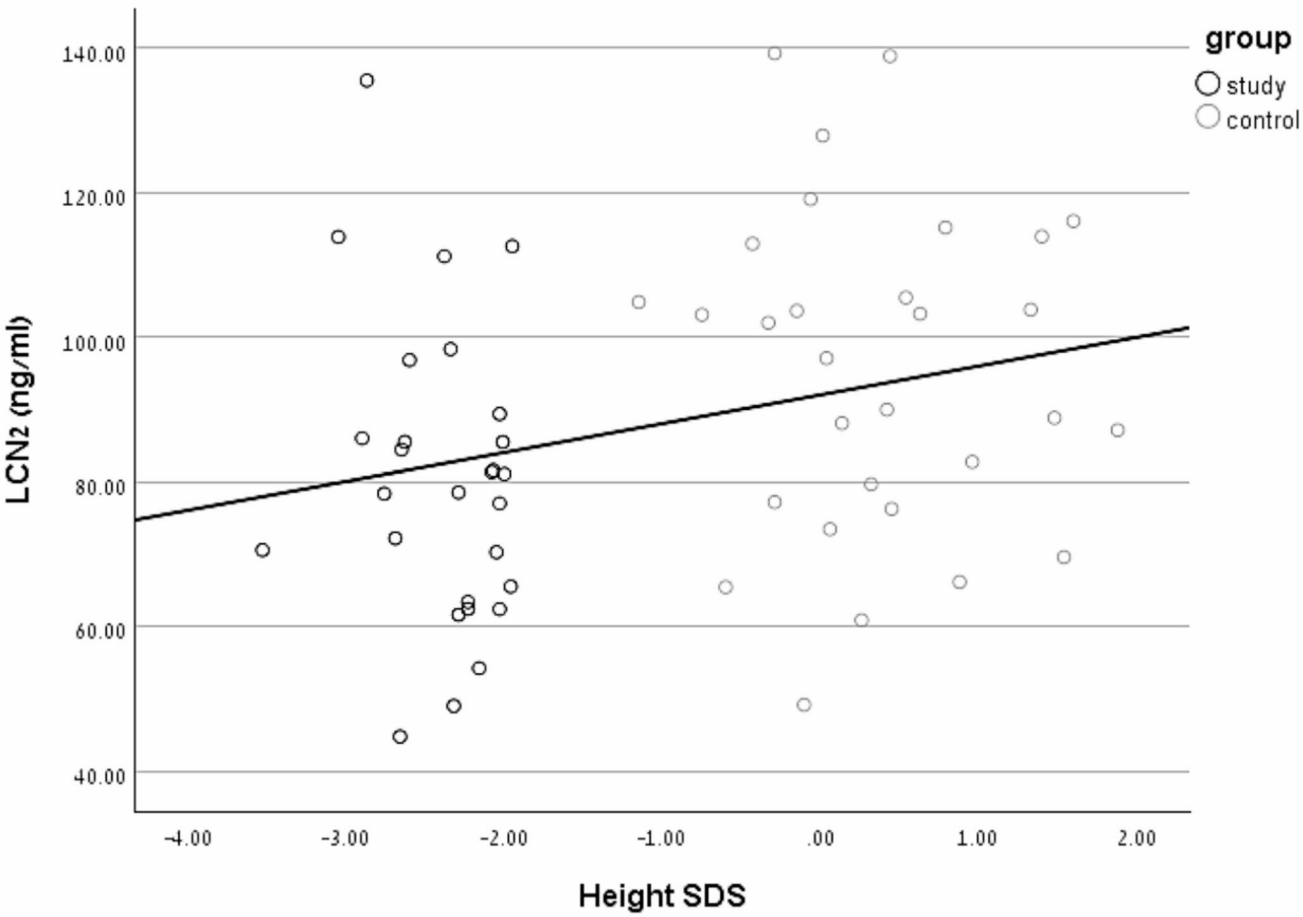
Correlation Between Height SDS and Serum LCN2 Levels. Scatter plot showing a positive correlation between height SDS and serum LCN2 levels (ng/mL) in children with ISS (*n* = 28) and controls (*n* = 30; Pearson’s *r* = 0.264, *p* = 0.045). Each circle represents an individual participant, with ISS (black) and controls (grey) distinguished

Height SDS was positively correlated with serum LCN2 levels across all participants (*r* = 0.264, *P* = 0.045, Fig. 1). No correlations were found between LCN2 levels and age, Δ height SDS, BMI SDS or IGF-1 SDS.

## Discussion

This study found that serum LCN2 levels were significantly lower in children with ISS compared to healthy controls with normal height, contrary to our hypothesis that LCN2 levels would be elevated in this population. We found a modest yet significant positive correlation between height SDS and LCN2 levels across all participants. Serum LCN2 levels were not correlated with any other clinical or biochemical characteristics, namely sex, age, BMI SDS, Δ height SDS, or IGF-1 SDS.

ISS likely represents a heterogeneous group of conditions, with overlapping genetic, hormonal and environmental influences. It is widely acceptable that genetic polymorphism explains a substantial number of children with ISS, but this is probably not the only cause. Chronic low-grade inflammation has been proposed as a potential mechanism impairing growth in ISS, as inflammation is known to disrupt the GH/IGF-1 axis by reducing levels of GH and IGF-1, decreasing hormone sensitivity, interference with downstream GH/IGF-1 signaling, dysfunction of IGF-binding proteins (IGFBPs), and diminished IGF bioavailability [15, 16]. Previous studies suggested that serum LCN2 levels are elevated in ISS, potentially reflecting an inflammatory state [2, 9]. However, our finding of lower LCN2 levels in children with ISS challenges this hypothesis, suggesting that systemic inflammation, as indicated by serum LCN2, may not be a primary driver of growth failure in this cohort.

There may be several explanations for these unexpected results. First, LCN2’s roles extend beyond inflammation, and include metabolic regulation with an effect on insulin sensitivity, appetite and energy expenditure [17]. While our two groups were well matched for age and sex, the ISS group had lower BMI SDS than controls, consistent with previous cohorts of children with ISS [18, 19], and lower rates of overweight and obesity. Although we found no correlation between BMI SDS and LCN2 levels, and a consistent difference in LCN2 levels between the groups after adjusting for BMI SDS, it is possible that there are metabolic differences between our study and control group which were not evaluated (insulin resistance, HOMA-IR) yet influenced our results. Second, our hypothesis was driven mainly by findings in animal models, most of which are related to increased local LCN2 expression at the growth plate, rather than serum LCN2 levels [7, 9]. Local expression of LCN2 in the growth plate and systemic LCN2 levels may, of course, be unrelated.

Limitations of this study include the small sample size (*n* = 28 ISS, *n* = 30 controls), which may limit generalizability, and requires future confirmation in larger cohorts. Our study lacks the analysis of other inflammatory markers (e.g., IL-6, TNF-α) to assess systemic inflammation. Finally, another limitation may be the collection of blood samples from members of the control group immediately after administration of anesthesia for minor surgery. To our knowledge, there are no specific studies assessing the immediate effect of anesthesia on circulating LCN2 levels. Of note, samples from both groups were collected in the morning after a night’s fast, to eliminate possible effects of eating and diurnal variation on LCN2 levels, as these parameters have been reported to affect results [20, 21].

## Conclusions

In this preliminary study in children with ISS, serum LCN2 levels were lower than in healthy controls and positively correlated with height SDS, suggesting that systemic low-grade inflammation, as reflected by LCN2 levels, is unlikely to be a primary cause of growth failure in ISS. These findings highlight the complex role of LCN2 in growth regulation. Further studies in larger cohorts are needed to confirm our results. Future research should consider evaluating fecal LCN2 levels and additional inflammatory cytokines to elucidate potential gut-related or inflammatory mechanisms in ISS.

## Data Availability

No datasets were generated or analysed during the current study.
